# Hypothyroidism Among Children and Adolescents With Nephrotic Syndrome in Mulago National Referral Hospital, Kampala, Uganda; a Cross-sectional Study

**DOI:** 10.21203/rs.3.rs-3021800/v1

**Published:** 2023-06-12

**Authors:** Maureen Tumwesige, Joseph Rujumba, Thereza Piloya, Carol Aujo

**Affiliations:** Makerere University College of Health Sciences; Makerere University College of Health Sciences; Mulago National Referral Hospital

**Keywords:** hypothyroidism, nephrotic syndrome, hypoalbuminemia, children, adolescents

## Abstract

**Background:**

Nephrotic syndrome (NS) is the commonest glomerular disease among children. It is characterized by heavy proteinuria and is a risk factor for hypothyroidism in the affected children. Hypothyroidism is of concern because it affects the physical and intellectual development of children and adolescents. This study sought to establish the prevalence and factors associated with hypothyroidism among children and adolescents with NS.

**Methods:**

A cross-sectional design was used to study 70 children and adolescents aged 1–19 years diagnosed with nephrotic syndrome and being followed up in the kidney clinic in Mulago National Referral Hospital. Questionnaires were used to collect patients’ socio-demographics and clinical information. A blood sample was taken for analysis for thyroid stimulating hormone (TSH) and free thyroxine (FT4), renal function tests and serum albumin. Hypothyroidism included both overt and subclinical forms. Overt hypothyroidism was defined as TSH level > 10 mU/L and FT4 < 10pmol/L, or FT4 < 10pmol/l with normal TSH, or TSH < 0.5mU/l. Sub-clinical hypothyroidism was defined as TSH ranging between 5 and10 mU/L with normal age appropriate FT4 levels. Urine samples were collected and taken for a dipstick examination. The data was analyzed using STATA version 14 and a p-value < 0.05 was considered as significant.

**Results:**

The mean age (standard deviation) of participants was 9 years (3.8). There were more males; 36 of 70 (51.4%). The prevalence of hypothyroidism was 23% (16/70 participants). Of the 16 children with hypothyroidism, 3 (18.7%) had overt hypothyroidism while 13 had subclinical hypothyroidism. Only low serum albumin, aOR 35.80 (confidence interval 5.97–214.69 and a p value of < 0.001) was associated with hypothyroidism.

**Conclusion:**

The prevalence of hypothyroidism among children and adolescent with nephrotic syndrome attending Mulago Hospital paediatric kidney clinic was 23%. Hypolbuminemia was found to be associated with hypothyroidism. Therefore, children and adolescents that have severely low levels of serum albumin should be screened for hypothyroidism and linked to endocrinologists for care.

## INTRODUCTION

Nephrotic syndrome (NS) is one of the most common renal glomerular diseases among children (1). The incidence of idiopathic NS alone has been reported to be 1.15–16.9 per 100,000 children and this has also been found to vary by ethnicity and region (2). The disease is classically defined by persistent massive range of proteinuria (≥ 40 mg/m^2^/hour or urine protein/creatinine ratio ≥ 200 mg/mL or 3 + protein on urine dipstick) and hypoalbuminemia(3). NS is a known risk factor for development of hypothyroidism as a result of the disease process and its treatment as well.

During the massive loss of protein in urine, proteins like thyroxine binding protein (TBG), albumin and thyroxine itself are also lost (4–6). These contribute to the development of primary hypothyroidism which is characterized by a low free thyroxine hormone (FT4) and a raised thyroid stimulating hormone (TSH) in an attempt to compensate for the loss. Furthermore, steroids which are the cornerstone of treatment of NS have also been reported to affect the release of TSH by the pituitary gland and can thus contribute to the development of central hypothyroidism (6).

Overt hypothyroidism can impair physical growth and intellectual development of children and adolescents if not diagnosed and treated in time (7, 8). Mario et al also documented that normalizing the thyroid status of children with NS can improve their response to treatment of the glomerular disease(5). Despite all this, there is no well-established protocol for screening of children and adolescents with NS for hypothyroidism (6).

In Uganda, proteinuric diseases in children have been reported to be eight times that which is seen in the United Kingdom with an incidence of 160 per a million population per year(9). There is however, paucity of data on the prevalence of hypothyroidism among children and adolescents with NS in Uganda where a higher incidence of proteinuric diseases are reported. The aim of this study was to therefore establish the prevalence and factors associated with hypothyroidism among children and adolescents with NS.

## METHODS

### STUDY DESIGN AND SETTING:

This cross-sectional study was carried out between February 2022 and July 2022 at Mulago national referral hospital (MNRH) children’s kidney clinic, in Uganda, East Africa. MNRH is Uganda’s largest and oldest national referral hospital and the services in this facility are free except certain laboratory investigations and specialized procedures. It is located in Kampala the capital city of Uganda but the hospital receives patients that are referred from different parts of the country. The paediatrics department was a children’s kidney ward where most patients with NS are first admitted before they are later followed up in the outpatient paediatric kidney clinic which runs on Mondays every week, alongside other specialized chronic diseases clinics. The clinic has a total of 100 children and adolescents registered with NS. All their medical information is stored in medical charts kept at the clinic. While NS on the kidney ward and clinic is managed in line with the Kidney Disease Improving Global Outcomes (3) guidelines, there is neither a guideline nor routine screening for hypothyroidism. However, growth monitoring is carried out at the clinic.

### STUDY POPULATION

All children and adolescents aged 1 to 19 years attending the Paediatric clinic at Mulago hospital with a documented diagnosis of NS in their files at time of enrollment into the clinic that was based on at least two of the following; of edema, hypoalbuminemia (≤ 2.5g/dl) and proteinuria ≥ + 3 or more on dipstick, irrespective of whether they were in remission or not. Those aged < 18 years whose parents and adolescents > 18 years who had given written informed consent and/children > 8yrs who assented were enrolled. Those that had a prior documented diagnosis of hypothyroidism before NS diagnosis were not eligible to take part in this study.

### STUDY PRODURE

During the study period, 71 patients with a diagnosis of NS attended the paediatric clinic at MNRH and by consecutive sampling, these were screened for eligibility; 70 children and adolescents were enrolled and one was excluded because they declined to provide consent and assent.

An interviewer administered questionnaire was used to collect data on the patient socio-demographics, clinical and NS disease characteristics, other risk factors for hypothyroidism (whether they used iodized salt, history of neck or brain surgeries/irradiations, other chronic medical conditions and medications used). Clinical history suggestive of hypothyroidism was also assessed; history of cold intolerance, constipation, fatigue, weight gain and menstrual irregularities, where applicable. Physical examinations were done and anthropometric measurements including weight in kilograms and length/height in centimeters that were taken using a weighing scale and stadiometer respectively. The patients’ files were reviewed for disease related factors that included the type of NS, cumulative dose of steroids, age at diagnosis of NS and the duration since the diagnosis of NS.

### LABORATORY INVESTIGATIONS

Blood samples were collected in plain red top vacutainers and transported to the laboratory within one hour of sample collection. The laboratory at MNRH was used to carry out the thyroid function tests (FT4, TSH), serum albumin and serum creatinine (from which the estimated glomerular filtration rate, eGFR, was calculated using Schwartz formula)(10). Thyroid function tests were measured using a fully automated COBAS 6000 ROCHE HITACHI machine from Germany which uses electrogenerated chemiluminescence (ECL) technology in which luminescence is produced during electrochemical reactions in solution and has been reported to be highly specific and sensitive (functional sensitivity at 0.01 mIU/L TSH)(11, 12).

Hypothyroidism in this study was defined as overt or subclinical forms. Overt hypothyroidism was defined as TSH level > 10 mU/L and FT4 < 10pmol/L or FT4 < 10pmol/l with normal TSH or TSH < 0.5mU/l. Sub-clinical hypothyroidism was defined as TSH ranging between 5 and10 mU/L with normal age appropriate FT4 levels that is: 1–5years [10–23.2pmol/L];6–10 years [10–28pmol/L] and 11–19 years [10–30pmol/L](13, 14).

Urine samples were collected from each participant and urinalysis was done. Urine protein was categorized as nil, trace, +, 2+, 3+.

### DATA MANAGEMENT

The sample size was calculated by Leslie Kish formula using a study that was done in Egypt by El-aal et al with estimated prevalence of hypothyroidism at 23.52% (15). This was adjusted to the available population according to the clinic records and a sample size of 70 patients was obtained. Consecutive sampling method was used.

Data was entered into an electronic database using Epidata version 3.1 software package with built-in quality control checks. It was then exported to Stata version 14.1 (STATA CORP, TEXAS USA for analysis. The continuous variables were summarized using means and standard deviations for normally distributed data. Simple Logistic regression analysis was used to test the association between hypothyroidism and independent variables. Crude odds ratio and its 95% CI was reported as measure of association at 5% level of significance and any variable that achieved a P value of < 0.05 was considered for multivariable analysis. Variables that attained a P value < 0.05 after multivariate analysis were considered statistically significantly and independently associated with hypothyroidism.

## RESULTS

**Figure 1:**
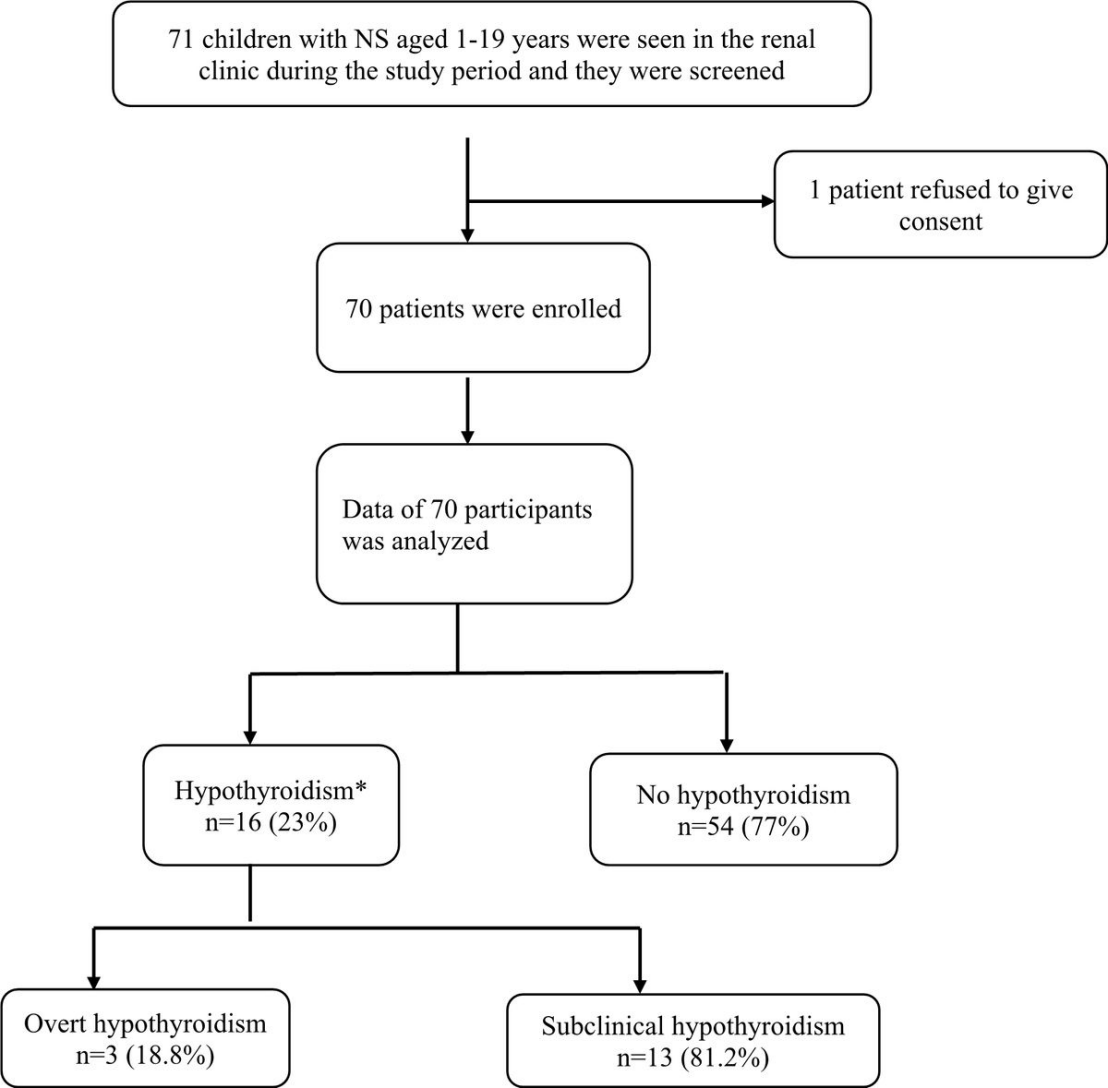
Study profile of children and adolescents with NS that were enrolled into this study. *those who were found to have hypothyroidism were linked to care in the paediatric endocrinology clinic

Seventy-one children and adolescents aged 1–19 years were screened during the study period from February 2022 to July 2022 and only 70 of these were enrolled. The prevalence of hypothyroidism was 23% (16 of 70 participants) with only 3 of the 16 children having overt hypothyroidism. The study enrollment profile is shown in Fig. 1 below.

### Baseline characteristics of patients enrolled

The mean age (standard deviation) of the children and adolescents that were enrolled was 9 years (3.8). The youngest was 2 years old while the oldest was 18 years old. The median age in years at the time of diagnosis of NS for the participants enrolled was 6 ± 3.3 IQR (3–9). The median (IQR) duration since the diagnosis of NS was made was 15.5 (8–48) months. Thirteen out of 70 patients (18.6%) resided in mountainous areas. There were 6 out of 70 patients with co-morbidities and these included sickle cell disease (in 3/6), HIV, hepatitis B and tetralogy of fallot. The cumulative dose of steroids was calculated from the patients’ records right from the time of enrollment into the renal clinic and the median (IQR) dose was 255.3 (110,490) mg/kg/day. It was found that few patients reported symptoms suggestive of hypothyroidism and none of the children had more than two symptoms. Cold intolerance was reported by 11/70 patients (15.7%), 24/70 (34.3%) reported weight gain, 25/70 (35.7%) reported fatigue and 10/70 (14.3%) patients reported constipation. None of the female adolescents reported menstrual irregularities. Of the patients that took part in the study, 43 out of 70 (61.4%) were in remission (had nil or trace proteins on urine dipstick). Their median serum creatinine levels in mg/dL (normal range 0.3–1 mg/dL) was 0.42(0.3–0.51) and the mean estimated GFR in mL/min/1.73 m^2^ was 143.4 ± 62.9. The mean serum albumin in g/dL (normal range 3.5–5.5 g/dL) was 3.4 ± 1.2. The median TSH in mU/L (normal range 0.7–4.5 mU/L) was 2.1 IQR (0.9–3.8) while the mean thyroxine level in pmol/L (normal range 10–28 pmol/L) was 15.6 ± 4.1. The rest of the characteristics are shown in Table 1.

### Description of the children with NS that had overt hypothyroidism

Three out of 16 participants with hypothyroidism had the overt form. One of these was a 3 year old male who had been first diagnosed with NS at the age of 2 years. He had no symptoms suggestive of hypothyroidism and had a cumulative dose of steroids (prednisolone) of 202mg/kg. At the time of enrollment into the study he had proteinuria + 2 on urine dipstick, serum albumin of 2.0 g/dL and a low FT4 of 7.49 pmol/L.

The second participant was a 4year old female who had been diagnosed with NS 2 months prior to enroll into the study. She was in remission at the time of enrollment and her prednisolone treatment was being tapered down gradually. Her cumulative dose of prednisolone was 78mg/kg and she reported to have cold intolerance. Her serum albumin was at 1.26g/dL and a low FT4 of 5.13 pmol/L.

The third participant was an 11 year old female who had only been diagnosed with NS a month prior to enrollment into the study. She was at Tanner stage I and reported cold intolerance and fatigue. Her cumulative dose of prednisolone was 82.5mg/kg. She had a proteinuria of + 4 on urine dipstick at the time of enrollment into the study, with serum albumin of 1.28g/dL and a low FT4 of 8.45pmol/L.

### Factors independently associated with hypothyroidism.

The factors that were found to be statistically significant at bivariate analysis (non-remission, presence of edema and reduced serum albumin) were then subjected to multivariate analysis and only reduced serum albumin < 2.5g/dl was found to be significantly associated with hypothyroidism, P value < 0.001 aOR 35.80(5.97,214.69). More information has been stated in Table 2.

## DISCUSSION

This study found a significantly high prevalence of hypothyroidism (23%) among children and adolescents with NS. However, we observed that majority had subclinical hypothyroidism with many being above 10 years of age. This can be explained by the fact that there are various physiological and physical changes that occur in the body of an adolescent that may result in increased secretion of TSH hence predisposing this age group to subclinical hypothyroidism (16). It is important to note that while subclinical hypothyroidism has been reported to spontaneously resolve in some cases, it can also persist and progress to overt hypothyroidism and hence there is need for continuous monitoring of the affected patients(17).

The prevalence found in this study was slightly lower than, the 33.3% prevalence of hypothyroidism reported by Marimuthu et al in a cross-sectional study done in India among children and adolescents aged 1–18 years with NS in the outpatient department(18). Although Marimuthu’s study had participants in a similar age group to this current study, with a mean age of 7.2 years (SD 3.9), the researchers probably reported a slightly higher prevalence because they only enrolled children and adolescents with steroid resistant NS(18). This type of NS is associated with longer duration of nephrosis therefore they may ultimately be losing more protein and thus loss of T4 and TBG causing hypothyroidism. (3). In this study however, we enrolled all patients with nephrotic syndrome of which the majority (67.6%, 55 out of 70) had steroid sensitive nephrotic syndrome and as such might be less likely to lose protein (including thyroglobulin, thyroxine and TSH) in urine. It is also important to note that autoimmune thyroid disorders have been reported to be higher in the Asian population and could have perhaps contributed to the high prevalence found by the researchers in this Indian study (19). Noteworthy, Marimuthu too found a similar proportion of children with subclinical hypothyroidism just like our study despite having a slightly younger population. (18). Therefore subclinical hypothyroidism may be the commonest form of hypothyroidism in NS population and whether this has long term clinical implications for the children and adolescents with NS may need further research through prospective studies.

This study found that patients with a reduced serum albumin were more likely to have hypothyroidism compared to those that had normal levels of the same. Children with NS lose protein in urine and among these proteins are thyroglobulin and serum albumin which are important carriers of the thyroid hormones but also the latter which are themselves protein in nature, are lost as well(4). Serum albumin also acts as a buffer of serum levels of thyroxine before hypothyroidism eventually occurs and once it is lost in urine together with thyroglobulin, the serum concentration of the thyroid hormones also decreases (4). This explains why the present study found that the children and adolescents that had hypoalbuminemia were more likely to have hypothyroidism. Although, it was not significant in this study, we also noted that majority of the participants with hypothyroidism were not in remission for the Nephrotic syndrome. This would further emphasize that prolonged proteinuria may ultimately lead to hypoalbuminemia with subsequent hypothyroidism.

These findings of hypoalbuminemia being associated with hypothyroidism are similar to the findings in a case-control study by Saffari et al which was conducted at a paediatric hospital in Qazvin, Iran. (20). The researchers reported a negative correlation between serum albumin and TSH levels in serum. TSH levels increase as a compensatory mechanism to the decrease in serum levels of thyroid hormones resulting from the loss of protein in urine among children and adolescents with NS.

Similarly, El-aal et al conducted a prospective study at Sohag University Hospital, Egypt over a one-year period among 51 children aged between 1 and 12 years old with NS and found that low levels of thyroid hormones and high levels of TSH (hypothyroidism) was significantly associated with low levels of serum albumin(15).

While universally most studies have reported a relationship between hypothyroidism and hypoalbuminemia, Jung et al reported contrary findings in a study conducted at Inje University Busan Paik Hospital, Korea. The researchers enrolled 31 children with NS between January 2001 and December 2017 where they compared their thyroid status during active nephrosis and in remission and they found no significant correlations between serum albumin and T4, TSH, or Free T4 levels(21). The researchers only found a negative correlation between T3 and serum albumin and they hypothesized that there were probably other mechanisms that could explain this other than the loss of protein in urine. However, the study by Jung et al had a very small sample size that may not have reached the power to detect the difference between those with low serum albumen and those with normal levels.

### STRENGTH AND LIMITATIONS

The study was conducted in the paediatric renal clinic in Uganda’s biggest national referral hospital that serves patients from different regions of the country and therefore the findings can be generalized to the rest of the population since there was a fair representation of all the regions of the country. The study also brings new information that shades light on the prevalence and factors associated with hypothyroidism among children with NS in Uganda, East Africa.

However, the study did not exclude autoimmune causes of hypothyroidism among the children with NS that we enrolled. The accessible population of children with NS in this study offered a limited sample size and hence the study may not have adequate power to detect other factors associated with hypothyroidism.

## CONCLUSION

The prevalence of hypothyroidism among children and adolescents with NS in MNRH is quite high as it affects 1 in 4 children. The factor that is associated with hypothyroidism among these children is hypoalbuminemia. Therefore, children and adolescents with NS that have hypoalbuminemia should be screened for hypothyroidism and the treating clinicians should endeavor to achieve and maintain normal albumin levels in these patients. Further studies that are preferably multi-center, with a larger sample size, are recommended to assess more factors that could be associated with hypothyroidism in NS.

## Figures and Tables

**Table 1:** Baseline characteristics of children and adolescents with NS that were enrolled in this study

Variable	Frequency (N = 70)	Percentage (%)
**Age category**		
< 5years	11	15.7
5–10 years	31	44.3
> 10 years	28	40
**Sex**		
Male	36	51.4
**Address**		
Urban	42	60.0
**Type of salt used**		
Iodized salt	58	82.9
Local and iodized salt	7	10.0
Local salt	4	5.7
No salt	1	1.4
**Weight for Age Z score (≤ 10yrs, n = 41)**		
WAZ − 2 to + 2 Z score-Normal	32	78.1
WAZ > + 2 Z score Obese/Overweight*	9	21.9
**Height for Age Z score**		
HAZ ≥ −2 to ≤ + 2-Normal	60	85.7
HAZ <-3 to <-2 –Moderate-severely stunted	10	14.3
**Type of nephrotic syndrome**		
Steroid sensitive NS	55	67.6.
Steroid resistant NS	11	15.7
Newly diagnosed**	4	5.7
**Degree of proteinuria on urine dipstick at enrollment**		
Nil and Trace	43	61.4
+ 1 and + 2***	6	8.6
+ 3 and + 4	21	30
**Presence of edema**	9	12.9
**Medication taken at enrollment**		
Prednisolone alone	30	41.1
Prednisolone and other drugs	13	18.8
Other drugs****	9	13.0.
None	18	26.1
**Laboratory results**		
Estimated GFR <90mL/min/1.73 m^2^	7	10.0
Reduced serum albumin (2.5g/dL)	17	24.3

* none of the children that were overweight/obese had edema,

** newly diagnosed were children and adolescents diagnosed in a period of less than 6 weeks and as such could not be classified as SSNS or SRNS,

*** did not meet nephrotic range proteinuria since they were on treatment,,

**** Other drugs included Tenofovir, abacavir, lamuvidine, efervarenz, captopril, folic acid, mycophenolate mofetil, tacrolimu

**Table 2:** Factors associated with hypothyroidism among children and adolescents with NS at Bivariate and multivariate analysis

Variable	Hypothyroidism		cOR, 95% CI	P value	aOR 95% CI	P value
	Yes (n = 16) (f,%)	No (n = 54) (f,%)				
**Age category**						
< 5years	5(31.3)	6(11.1)	5.63(1.15,2744)	0.053		
5–10 years	4(25.0)	27(50.0)	1.00			
> 10 years	7(43.8)	21(38.9)	2.25(0.58,8.72)	0.241		
**Sex**						
Male	10(62.5)	26(48.2)	1.79(0.57,5.64)	0.316		
Female	6(37.5)	28(51.8)	1.00			
**Address**						
Urban	12(75.0)	30(55.6)	2.40(0.69,8.39)	0.171		
Rural	4(25.0)	24(44.4)	1.00			
**Comorbidity known**						
Yes	14(87.5)	50(92.6)	1.79(0.29,10.78)	0.527		
No	2(12.5)	4(7.4)	1.00			
**Age of the child at time of diagnosis**						
< 5 years	7(43.7)	18(33.3)	0.58(0.13,2.71)	0.492		
5–10 years	5(31.3)	30(55.6)	1.00			
> 10 years	4(25.0)	6(11.1)	0.58(0.13,2.71)	0.086		
**Type of nephrotic syndrome**						
Steroid sensitive NS	11(68.8)	44(81.5)	1.00		1.00	
Steroid resistant NS	2(12.5)	9(16.7)	0.89(0.17,4.72)	0.89	0.71(0.03,14.29)	0.822
Newly diagnosed	3(18.8)	1(1.8)	12.0(1.13,126.79)	0.039	2.63(0.04,158)	0.664
**Remission status at enrollment**						
Yes	5(31.2)	38(70.4)	1.00		1.00	
No	11(68.8)	16(29.6)	**5.33 (1.56, 17.48)**	**0.007**	3.56(0.52,24.45)	0.196
**Medication taken at enrollment**						
Prednisolone alone	7(43.8)	22(41.5)	5.41(0.61,48.27)	0.131		
Pred and other drugs^a^	4(25.0)	9(16.9)	7.55(0.73,78.08)	0.091		
None	1 (6.2)	18(32.1)	1.00			
**Edema**						
Yes	6(37.5)	3(5.6)	**10.2(2.18,47.71)**	**0.003**	1.29(0.11,15.11)	0.836
No	10(62.5)	51 (94.4)	1.00		1.00	
**Laboratory findings**						
**Estimated GFR**						
Normal eGFR	14(87.5)	49(90.7)	1.00			
Reduced eGFR	2(12.5)	5(9.3)	1.40(0.24,8.01)	0.705		
**Serum albumin**						
Normal (NR3.5–5.5g/dL)	3(18.7)	50(92.6)	1.00		1.00	
Reduced(≤ 2.5g/dL)	13(81.3)	5(7.4)	**54.17(10.75,272.76)**	**0.000**	**35.80(5.97,214.69)**	**0.000**

a Prednisolone and other drugs which included captopril, tacrolimus, mycophenolate mofetil

## Data Availability

The dataset that was generated during this study is not publicly available because we did not obtain consent from all the participants to publish raw data. It can however be availed by the corresponding author upon reasonable request.

## Abbreviations

eGFR: estimated glomerular filtration rate
FT4: Free thyroxine
KDIGO: Kidney Disease Improving Global Outcomes
MNRH: Mulago National Referral Hospital
NS: Nephrotic syndrome
TSH: Thyroid Stimulating Hormone
